# Role of c-Jun N-terminal Kinase (JNK) in Obesity and Type 2 Diabetes

**DOI:** 10.3390/cells9030706

**Published:** 2020-03-13

**Authors:** Justin Hou Ming Yung, Adria Giacca

**Affiliations:** 1 Department of Physiology, University of Toronto, Toronto, ON M5S 1A8, Canada; justin.yung@mail.utoronto.ca; 2 Department of Medicine, University of Toronto, Toronto, ON M5S 1A8, Canada; 3 Institute of Medical Science, University of Toronto, Toronto, ON M5S 1A8, Canada; 4 Banting and Best Diabetes Center, University of Toronto, Toronto General Hospital, Toronto, ON M5S 2C4, Canada

**Keywords:** c-Jun N-terminal kinase, JNK, type 2 diabetes, inflammation, obesity, insulin resistance, lipotoxicity, glucotoxicity

## Abstract

Obesity has been described as a global epidemic and is a low-grade chronic inflammatory disease that arises as a consequence of energy imbalance. Obesity increases the risk of type 2 diabetes (T2D), by mechanisms that are not entirely clarified. Elevated circulating pro-inflammatory cytokines and free fatty acids (FFA) during obesity cause insulin resistance and ß-cell dysfunction, the two main features of T2D, which are both aggravated with the progressive development of hyperglycemia. The inflammatory kinase c-jun N-terminal kinase (JNK) responds to various cellular stress signals activated by cytokines, free fatty acids and hyperglycemia, and is a key mediator in the transition between obesity and T2D. Specifically, JNK mediates both insulin resistance and ß-cell dysfunction, and is therefore a potential target for T2D therapy.

## 1. Obesity and Type 2 Diabetes

The prevalence of obesity has substantially increased in the past few decades, now being described as a global epidemic. As food that is high in caloric content is becoming more available and technology becomes more efficient, there is a shift towards increased energy intake and reduced energy expenditure. This imbalance ultimately leads to the excessive accumulation of fat [1]. Obesity is associated with increased risk of developing health problems, such as type 2 diabetes (T2D) [2], cardiovascular disease, liver disease, cancer [3], and even neurodegenerative diseases, in particular Alzheimer’s disease [4,5].

Not surprisingly, as a result of the obesity epidemic, there is also an increasing incidence of T2D, a disease that is characterized by insulin resistance and a relative defect in insulin secretion [6]. Most obese subjects who have insulin resistance are able to compensate for it by increasing insulin released by ß-cells of the pancreas. However, once ß-cells start malfunctioning, the fall of compensatory insulin secretion will cause hyperglycemia [7].

**Figure 1:**
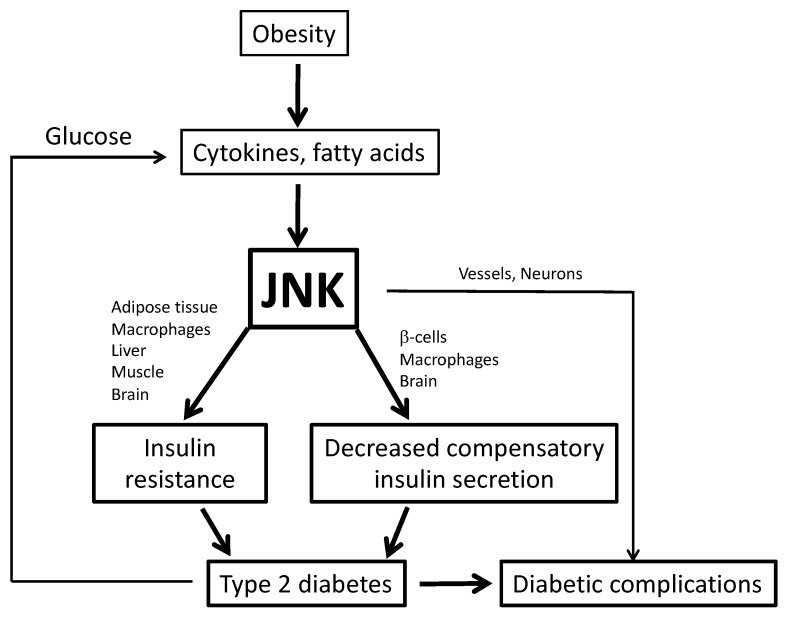
c-Jun N-terminal kinase (JNK) is an inflammatory kinase that is ubiquitously expressed. During obesity, a low-grade chronic inflammatory disease, there are elevated cytokines and free fatty acids, which can activate JNK in various tissues. Activation of JNK is implicated in obesity-induced insulin resistance and decreased compensatory insulin secretion, both of which are key features of type 2 diabetes (T2D). During T2D, there are elevated glucose levels in plasma, which also contribute to the activation of JNK. Activation of JNK is also associated with diabetic complications.

Although the mechanisms linking obesity and the development of T2D are complex and somewhat controversial, the role of inflammatory kinases is currently being investigated as a major factor in both insulin resistance and ß-cell dysfunction. This review will focus on the role of c-Jun N-terminal kinase (JNK) in T2D, as it has been found that the activity of this kinase is elevated during obesity and diabetes and this kinase can induce insulin resistance and ß-cell dysfunction (Figure 1) [8]. 

## 2. c-Jun N-terminal Kinase (JNK)

JNK is a serine/threonine kinase, which is part of the mitogen activated protein kinase (MAPK) family and is an important protein present in all cells for mounting appropriate responses to stress. There are 3 *Jnk* genes that generate at least 10 isoforms through splicing: *Jnk1* and *Jnk2* are ubiquitously expressed [9], while the expression of *Jnk3* is specific to the brain, testis, heart, and the pancreas [10]. The result of JNK activation largely depends on the context and duration of activation. Transient JNK activity could result in proliferation, whereas prolonged activation could trigger cell death [10].

In order for JNK to be activated, there must be two phosphorylation events. There is a Thr-Pro-Tyr motif within the activation loop, where the Thr/Tyr phosphorylation events must occur for full activation. Several MAPK kinases can catalyze the phosphorylation events, while the MAPK phosphatases catalyze the removal of the phosphate groups, providing positive and negative regulation of JNK activation, respectively [11].

JNK is considered to be one of the effectors of the MAPK signaling pathway, as it is the last kinase to be activated within the MAPK/JNK pathway. Other effectors of the MAPK signaling pathway are p38 MAPK, ERK1/2, and ERK5 [12]. When the MAPK signaling pathway is activated by an extracellular or intracellular signal, the first kinase, MAP3K, is activated. MAP3K is a common name for the different kinases that make up the upstream kinases that are part of the MAPK signaling pathway and can initiate all MAPK signaling. This group of kinases will phosphorylate downstream kinases, collectively termed as the MAP2Ks, on Ser and Thr residues for activation. Once active, MAP2K will Thr/Tyr phosphorylate and activate MAPK, thus effectively transmitting an extracellular signal into an appropriate cellular response [13]. By having this step-by-step progression, various signals can be integrated into cellular responses, which can provide multiple points of regulation, as well as points of crosstalk with other signaling pathways within the cell [11].

Specific to the JNK pathway, TAK1, MEKK1, MEKK4, ASK1, and MLK are all considered MAP3Ks, as these kinases are first to respond to various inputs that eventually activate JNK. For instance, TAK1 senses polyubiquitination events at the tumor necrosis factor (TNF) receptor, while MEKK4 can sense DNA damage. The activation of these kinases will subsequently activate the downstream MAP2Ks MKK4 and MKK7, the most important for the JNK pathway. Some of the MAP3Ks mentioned above preferentially activate MKK4, while others activate MKK7. These kinases then phosphorylate JNK on Tyr185 and Thr183 respectively to activate it [13]. Typical targets of JNK are transcription factors such as c-Jun, ATF2, p53, and c-Myc. c-Jun is also part of the AP-1 transcription factor family, thus when JNK increases c-Jun phosphorylation, JNK also enhances AP-1 activity. Additionally, JNK can directly phosphorylate other proteins within the cell, such as Bcl-2, Bcl-xL, Bim and BAD, all of which are members of the Bcl2 family, that have their own functions in controlling apoptosis [14].

For example, the activation of TNF receptor by tumor necrosis factor α (TNFα), a common cytokine elevated during obesity and T2D, activates TAK1, which preferentially activates MKK7, and together with MKK4 will cause the activation of JNK. Mono-phosphorylation of JNK by MKK7 on Thr183 is sufficient to stimulate JNK activity [15], however phosphorylation on Tyr185 by MKK4 is required for full activation of JNK [16]. It is still unclear as to how MKK4 is activated in the context of cytokine exposure; one possibility lies in the fact that PKC-induced phosphorylation of JNK may sensitize JNK to MKK4/7 action [17].

## 3. Role of Inflammation and JNK in Metabolic Disease

In recent years, inflammation has been increasingly researched in the context of metabolic disorders. Obesity can be described as a chronic, low-grade inflammatory disease. The first major link made between inflammation and obesity identified the pro-inflammatory cytokine TNFα: tissues obtained from obese humans, as well as the adipose from murine models of obesity, had elevated TNFα expression. Further studies found that TNFα can perturb insulin signaling, causing insulin resistance. In addition, animals without the TNF receptor had restored insulin sensitivity, strongly supporting the role of inflammation in obesity and its associated metabolic disorders [18]. This was confirmed by further investigation demonstrating that adipose tissue isolated from obese humans and mouse models had upregulation of pro-inflammatory cytokine expression [19]. However, the processes responsible for inflammation are still not completely understood. 

### 3.1. Adipose Tissue Inflammation

One tissue that is significantly altered during obesity is the white adipose tissue (WAT). WAT have two main components: adipocytes and the stromal vascular fraction (SVF). The SVF includes the extracellular matrix, along with multi-potent stem cells, pre-adipocytes, and immune cells. During obesity and the expansion of fat mass, one theory suggests that there is reduced blood flow and access of nutrients, leading to hypoxia within WAT. Due to the lack of oxygen, the hypoxia-inducible factor-1α (HIF-1α) and nuclear factor kappa B (NFκB) signaling pathways can become active, resulting in increased expression of pro-inflammatory adipokines [20]. Especially when there is adipocyte death via apoptosis or necrosis, there is elevated secretion of monocyte chemoattractant protein 1 (MCP1) and TNFα by adipocytes to recruit macrophages and other immune cells. These dying cells can also cause expansion of immune cells that reside in adipose tissue, increasing the inflammatory response within this tissue. Recruited and activated immune cells can then secrete cytokines that will disrupt local adipocyte physiology, lipid storage and breakdown [19]. Hypoxia provides one possible explanation as to how inflammation can develop within the adipose tissue during obesity. Other theories include excess hypertrophy of adipocytes, leading to an increased number of dysfunctional adipocytes which undergo apoptosis and contribute to inflammation [21]. 

Expansion of immune cells, especially macrophages, within the adipose tissue is a major event that occurs during obesity-induced inflammation. During a non-obese state, resident adipose tissue macrophages (ATM) are in a balance between their M1 and M2 phenotype. The M1 phenotype is characterized as the pro-inflammatory state, as these macrophages secrete pro-inflammatory cytokines, while the M2 phenotype, an anti-inflammatory state, can protect against insulin resistance by secreting anti-inflammatory cytokines such as interleukin-10 (IL-10). During obesity, this balance is disrupted, and as such, there is polarization of ATM into their M1 phenotype [22]. This conversion in phenotype has been associated with obesity-mediated adipose tissue inflammation [23]. Pro-inflammatory M1-macrophages are key factors in generating inflammatory molecules, such as TNFα, IL-1ß and IL-6, that cause insulin resistance both in adipose tissue and other insulin-sensitive tissues [24]. In fact, these M1 macrophages that are activated within the adipose tissue have been identified to be a major source of circulating TNFα and IL-6 during obesity, insulin resistance, and T2D [25]. These cytokines can bind to their receptors on the cell surface of adipocytes and further elevate the expression of pro-inflammatory adipokines and can continually drive adipose tissue inflammation [26]. Downstream of receptor activation is JNK, which, when activated, can cause increased Ser/Thr phosphorylation of IRS1/2, reducing their Tyr phosphorylation by the activated insulin receptor and thus causing insulin resistance [26,27]. Insulin resistance in insulin-sensitive tissues will be further discussed in the following sections.

The release of pro-inflammatory cytokines by macrophages also plays a role in a feed-forward cycle, because these cytokines cause the recruitment of more macrophages and circulate in blood to propagate the inflammation to other insulin sensitive tissues [26]. The removal of macrophages using liposome clodronate in mice given high-fat diet or in *ob/ob* mice improved whole-body insulin sensitivity and glucose tolerance [22,28,29]. 

The aforementioned pro-inflammatory cytokines that cause JNK activation within adipocytes can also cause reduced triglyceride storage and increased lipolysis. This is one reason why during obesity there is elevated free fatty acids (FFA) in plasma, although the main driver of the enhanced adipose tissue lipolysis is insulin resistance [30]. Cytokines can negatively impact the transcription factor peroxisome proliferator-activated receptor γ (PPARγ). This factor is important for the process of adipocyte maturation and proper triglyceride synthesis [19,31]. For example, TNFα can decrease PPARγ expression [32]. This limits further adipose tissue expansion, thus favoring ectopic fat accumulation. FFA can activate the JNK and NFκB pro-inflammatory pathways that cause the increased expression of pro-inflammatory cytokines, such as TNFα and IL-6 [33,34] via PKC and ROS. In addition, FFA, in particular saturated FFA, can cause ER stress that also activates the JNK and NFκB pathways [35,36,37]. Palmitate generates the sphingosine backbone of ceramides, which are FFA esterification products [38]. Ceramides not only directly interfere with the insulin signaling pathway by inhibiting AKT (discussed in a later section), but can also activate the JNK pathway [39,40]. Saturated FFA, such as palmitate, can activate innate immunity receptors such as toll-like receptors 2/4 (TLR2/4) that are found on the cell surface of adipocytes and macrophages, which can activate the JNK and NFκB pathways [41,42,43,44]. Furthermore, the activation of TLR4 has been shown to increase ceramide synthesis through an IKK-dependent mechanism [39]. 

### 3.2. Ectopic Fat Accumulation on Insulin Resistance and ß-Cell Dysfunction

As discussed above, inflammation in the adipose tissue causes changes in the metabolism of lipids. In an insulin resistant state, there are elevated triglyceride levels in plasma, due to the increased availability of FFA precursors for very-low density lipoprotein (VLDL) secretion and to reduce stimulation of lipoprotein lipase by insulin, resulting in the inefficient removal of lipids from circulation. In addition, the elevation of triglycerides in plasma is also a result of increased lipogenesis in the liver and the dysregulation of VLDL export from the liver, due to hepatic insulin resistance [45]. Elevated plasma FFA and triglycerides can be taken up by fat, and can accumulate within insulin-sensitive tissues. This is called ectopic fat accumulation, which contributes to insulin resistance within these organs. For example, the accumulation of intramyocellular lipids (IMCL) has been linked to the development of skeletal muscle insulin resistance [46]. Such lipid accumulation can be contributed to by the impairment of fat oxidation, due to fat-induced mitochondrial dysfunction. Fatty acid overload of mitochondria is thought to generate products of incomplete oxidation, which include ROS. This not only induces insulin resistance by activating inflammatory kinases, but also causes mitochondrial damage [47]. Once mitochondrial oxidation is reduced, fatty acids are diverted to esterification and ectopic fat accumulation, which generates toxic esterification products such as DAG and ceramides [48,49]. Several studies have found an association between reduced mitochondrial function in muscle samples obtained from patients who are obese or have T2D, and insulin resistance [50,51,52,53,54,55]. Furthermore, two important studies found that gene expression and activity of peroxisome proliferator-activated receptor-gamma coactivator (PGC)-1α, a transcriptional coactivator that stimulates mitochondrial function [56], is reduced in patients with T2D or in those who have a family history of T2D [57,58]. This solidified the concept that reduced mitochondrial function in skeletal muscle plays a role in the development of insulin resistance. In relation to insulin secretion, although fat in the exocrine pancreas is a poor marker of intra-islet fat accumulation, two studies found a correlation of increased pancreatic fat content during obesity, with declined ß-cell function [59,60]. Accumulation of intra-islet fat can cause islet inflammation by the same mechanisms as in insulin-sensitive tissues, thus impairing glucose-stimulated insulin secretion and contributing to ß–cell death [7]. 

## 4. Role of JNK in Insulin Resistance

As mentioned earlier, insulin resistance is defined as the inability of insulin-sensitive tissues, such as skeletal muscle, liver, adipose, and brain, to respond appropriately to insulin. The insulin signaling pathway is very similar among all these tissues: it starts with insulin binding to the membrane-bound tyrosine kinase receptor. Once insulin binds, the receptor undergoes a conformational change, allowing for the autophosphorylation event on the intracellular tyrosine kinase domains of the receptor. When this domain is phosphorylated, it provides a docking site and a signal for the recruitment of the insulin receptor substrate 1/2 (IRS1/2) proteins. These proteins are then phosphorylated on tyrosine residues by the active tyrosine kinase domain of the insulin receptor. This event will subsequently activate the PI3K-AKT pathway, which is known to mediate metabolic effects [61]. Downstream of AKT activation, what differs are the tissue-specific effects of insulin action. In skeletal muscle, insulin causes membrane translocation of GLUT4 transporters, allowing for glucose uptake, and stimulates glycogen synthesis. In the liver, insulin promotes glycogen synthesis and the expression of glycolytic enzymes, reduces the expression of gluconeogenic enzymes, and increases fatty acid and triglyceride synthesis. In adipose tissue, insulin enhances glucose uptake by increasing the translocation of GLUT4 transporters to the cell surface, and increases fatty acid and triglyceride synthesis [62]. In the brain, particularly the hypothalamus, insulin plays important roles in energy homeostasis, decreasing food intake and increasing energy expenditure. It increases energy expenditure by elevating body temperature and sympathetic nervous system activity [63]. As an anorexigenic hormone, insulin binds to its receptors within the arcuate nucleus of the hypothalamus, causing reduced expression of orexigenic neuropeptides such as neuropeptide Y (NPY) and Agouti-related peptide (AgRP) to cause cessation of hunger and reduce food intake [64]. Insulin action within the hypothalamus also affects peripheral glucose metabolism. For instance, insulin signaling within the AgRP-expressing neurons of the hypothalamus can reduce gluconeogenesis via vagal inputs to the liver [65,66,67,68]. PI3K is activated within the hypothalamus, increasing concentrations of PIP3, which activates the ATP-sensitive K^+^ channel, causing hyperpolarization (thus inactivation) of the vagal nerve that innervates the liver. This reduces acetylcholine (ACh) signaling within the Kupffer cells, due to lack of ACh binding to the α7-nicotinic ACh receptor (α7-nAChR) [69]. Decreased α7-nAChR activation, in turn, stimulates the release of IL-6 from the Kupffer cells, which activates STAT3 signaling in hepatocytes, causing a reduction in gluconeogenic gene expression and hepatic glucose production [70]. Insulin signaling within non-neuronal cells have also been implicated in peripheral glucose homeostasis. Mice with ablation of insulin receptor in hypothalamic astrocytes exhibited glucose intolerance, as assessed by a glucose tolerance test [71].

Another pathway that is activated when insulin binds to its receptor is the MAPK pathway, which is responsible for mediating mitogenic effects. When the same tyrosine kinase domain is phosphorylated at the receptor, the substrate Shc, which unlike IRS1/2, does not bind to PI3K, is also phosphorylated, allowing the adaptor protein GRB2 to bind. Once bound, Ras guanylnucleotide exchange factor (SOS) is recruited to the plasma membrane, in close proximity to Ras, so that it can exchange GDP for GTP on Ras itself. Ras-GTP will then activate the family of MAP3K Ser/Thr kinases, which can phosphorylate and activate downstream MAP2K and MAPK proteins [72]. For example, insulin can stimulate cellular proliferation when the MAP3K Raf is activated. Raf activation will subsequently activate the MAP2Ks MEK1/2, which then phosphorylate the MAPK effectors ERK1/2, p38, or JNK [73,74,75]. Thus, insulin can activate JNK. However, insulin resistance does not decrease JNK activation, because insulin resistance is selective for the PI3K/AKT pathway. Indeed, several studies have demonstrated the concept of “selective insulin resistance”, where there is reduced activation of the PI3K/AKT pathway, whereas the MAPK pathway is unaffected [76,77]. During a state of insulin resistance and hyperinsulinemia, the metabolic PI3K/AKT pathway is down-regulated, due to Ser/Thr phosphorylation of IRS by PKC and inflammatory kinases, such as IKKß and indeed JNK, which impedes insulin-induced IRS tyrosine phosphorylation [78,79], and the effect of SOCS, which also impede IRS tyrosine phosphorylation [80]. However, the mitogenic Ras/Raf/MAPK (JNK) pathway is unaffected during insulin resistance or is even upregulated by the compensatory hyperinsulinemia, because Shc Tyr phosphorylation by insulin is not affected [81]. 

**Figure 2:**
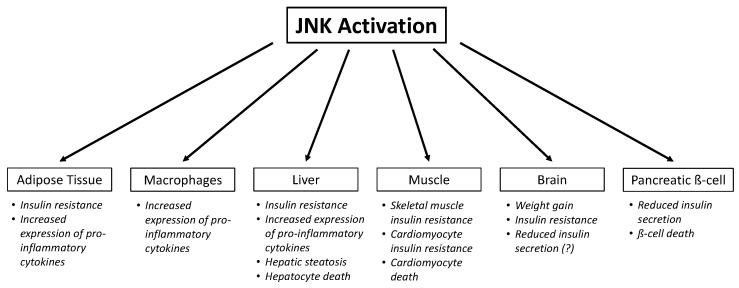
Chronic JNK activation in different tissues leads to different effects. Since JNK has widespread action in different tissues, it is an important contributor of obesity-induced insulin resistance and T2D. In adipocytes, JNK activation causes adipocyte insulin resistance and the increased expression of pro-inflammatory cytokines such as TNFα, IL-6 and IL-1ß. In macrophages, JNK activation also causes increased expression of pro-inflammatory cytokines that promotes M1-polarization, the pro-inflammatory phenotype, and subsequent recruitment of more M1 macrophages. In the liver, JNK activation causes hepatic insulin resistance, steatosis, and hepatocyte death. In myocytes, JNK activation causes skeletal muscle insulin resistance and cardiomyocyte insulin resistance and cardiomyocyte death. In pancreatic ß-cells, JNK activation reduces insulin secretion and ß-cell death. In the brain, particularly the hypothalamus, JNK activation causes central insulin resistance, which impacts energy homeostasis (mainly inducing food intake and weight gain), brain regulation of glucose production, and might also reduce insulin secretion by β-cells.

Obesity greatly contributes to the development of insulin resistance, due to elevated cytokines and FFA, and reduced adiponectin. JNK is an important contributor of obesity-induced insulin resistance and T2D, because of its widespread action in different tissues (Figure 2). Studies in whole body JNK1-null mice indicated that when mice are subjected to a high-fat diet (HFD), JNK1 is the most important isoform associated with obesity and plays a part in causing insulin resistance in insulin-sensitive tissues. Mice with systemic ablation of JNK1 are protected from HFD-induced inflammation and insulin resistance. These knockout (KO) mice on the same high-fat diet were also resistant to developing obesity, because of reduced adiposity, likely due to the JNK effect on hypothalamic inflammation affecting central regulation of energy balance by insulin and leptin. These effects were correlated with a reduction in the serine-307 phosphorylation of IRS1, which prevents proper activation of the insulin signaling pathway [10]. However, as a Ser/Thr kinase, it is likely that JNK1 phosphorylates multiple sites on IRS1/2, since subsequent studies found that a serine to alanine mutation on residue 307 (S307A mutation) on IRS1, a mutation that prevents phosphorylation by JNK1, does not protect mice from HFD-induced insulin resistance [82], and that only with multiple serine mutations into alanine at position 302, 307 and 612 was insulin sensitivity restored in skeletal muscle [83]. Additionally, JNK1 phosphorylates IRS2 on Ser/Thr residues, which could also explain why mutation on one IRS1 residue did not prevent obesity-induced insulin resistance. Whole-body JNK2-null mice did not share the same protection as JNK1-null mice; these mice even displayed increased obesity and insulin resistance. This was due to overcompensation by JNK1, which contributes to insulin resistance [10]. 

### 4.1. Adipose Tissue

Adipose tissue is an important energy storage organ that can convert excess nutrients into triglycerides. It is also an important endocrine tissue, as it also releases a number of hormones that are important for metabolism. In healthy individuals, when insulin binds to its receptors on the cell surface of adipocytes, it enhances anabolic processes, such as triglyceride formation and storage, and reduces lipolysis. Similar to skeletal muscle and liver, JNK1 has been found to be upregulated in adipocytes during obesity [8]. 

Several in vitro studies using the 3T3-L1 adipocyte cell line concluded that JNK plays a role in mediating FFA-induced insulin resistance, and inhibiting JNK has beneficial effects in restoring insulin sensitivity [84,85]. One in vivo study examined whether adipocyte-specific JNK1 ablation would be beneficial for adipose tissue insulin sensitivity and found that adipocyte-specific JNK1-null mice were protected from HFD-induced insulin resistance, at the level of adipose tissue and surprisingly, also at the level of the liver. In the same study, IL-6 was the only pro-inflammatory cytokine found to be elevated in wildtype (WT) mice on HFD, compared to adipocyte-specific JNK1-null mice on HFD, and its administration in these JNK1-null mice abolished their protection from HFD-induced hepatic insulin resistance [86]. Thus, IL-6 is proposed to have dual effects in the liver (see the regulation of glucose production by brain insulin described above).

### 4.2. Macrophages

It is clear that macrophages play a role in mediating inflammation during a state of obesity to cause insulin resistance and potentially cause ß-cell dysfunction. Not only can macrophages be recruited and polarized into a pro-inflammatory state by cytokines, but they can be directly activated by saturated FFA, such as palmitate, that are released by the expanding adipose tissue. Pattern recognition receptors, which are the receptors of innate immunity, initiate inflammatory processes in response to foreign pathogens, but more recently it has been established that they can also be activated by saturated FFA. For example, TLR4 is activated by saturated FFA such as palmitate [42]. When activated, recruitment and activation of two different signaling pathways, either TRAM/TRIF/RIP1 or TIRAP/MyD88/IRAK/TRAF6, will cause the activation of TAK1, a kinase that will eventually activate NFκB and JNK, and will enhance IL-1ß, TNFα, and IL-6 secretion to induce further insulin resistance [87,88]. In fact, macrophages exposed to palmitate displayed prolonged activation of JNK1 and significantly elevated expression of pro-inflammatory cytokines TNFα and IL-6, while macrophages with JNK1 ablation had significantly reduced expression of these cytokines [89]. In addition, macrophage-specific JNK1/2-null mice on a high fat diet were protected from insulin resistance, as seen by elevated AKT activation in insulin-sensitive tissues, as compared to wild-type mice on the same diet. In addition, the macrophage-specific JNK-null mice were protected from hyperglycemia and hyperinsulinemia compared to WT mice on the same diet. Furthermore, the macrophage-specific JNK-null mice did not have the same extent of M1-macrophage accumulation and pro-inflammatory cytokine expression in adipose tissue, as compared to WT mice. However, it was observed that the mice in these two groups did not display differences in plasma FFA levels, which suggests that macrophage-specific JNK does not play a big role in obesity-induced lipolysis. Altogether, this suggests that JNK is required in macrophages for mediating the effects of obesity-induced inflammation and insulin resistance [90].

### 4.3. Skeletal Muscle

In healthy, normal glucose tolerant (NGT) individuals, skeletal muscle is responsible for a large portion of glucose utilization in the body. After a meal, insulin is released from ß-cells and will cause most of the glucose to be taken up by skeletal muscle. However, during an insulin resistant state, uptake of glucose is not as efficient, due to perturbations of the insulin signaling pathway. 

There is debate in the literature on whether JNK contributes to insulin resistance specifically in skeletal muscle. In one study, JNK1 was knocked down in skeletal muscles using the Cre-lox system and the authors found that muscle-specific JNK1-null mice were protected from obesity-induced skeletal muscle insulin resistance. Although these KO mice did not display overall improved glucose tolerance compared to control mice, the beneficial effects towards skeletal muscle insulin sensitivity were confirmed with both insulin tolerance tests and hyperinsulinemic-euglycemic clamps. These KO mice had increased insulin signaling in skeletal muscle due to reduced phosphorylation on serine residues on IRS1/2 [91]. In line with the results from this study, another group generated a fusion protein that made JNK1 constitutively active, and electroporated this protein into skeletal muscle in mice, and found that the constitutively active JNK1 in muscle reduced glucose uptake stimulated by insulin [92]. Contrasting the results of these two studies, Pal et al. reported no change in glucose homeostasis or insulin sensitivity under obese conditions in mice that expressed constitutively active JNK1 specifically in skeletal muscle as well as in muscle-specific JNK1-null mice. However, these authors performed glucose and insulin tolerance tests but did not assess insulin resistance with the gold standard method, which is the hyperinsulinemic-euglycemic clamp [93]. 

### 4.4. Liver

The liver is an extremely important metabolic regulatory organ, as it is responsible for both producing glucose during fasting and storing glucose during times of energy excess. Unlike skeletal muscle, insulin does not cause increased transport of glucose into hepatocytes; rather it increases glycogen synthesis and lipid accumulation, and decreases gluconeogenesis. 

One of the most common liver diseases associated with obesity is nonalcoholic fatty liver disease (NAFLD), which is characterized by fat accumulation, insulin resistance, and in its more severe forms, also known as nonalcoholic steatohepatitis (NASH), by hepatocyte apoptosis [94]. Elevated cytokines and FFA in an obese state can promote insulin resistance through the mechanisms described above, and ultimately induce hepatic fat accumulation. Fat accumulation in the liver can cause insulin resistance but insulin resistance does not cause fat accumulation because insulin increases lipogenesis and fat esterification in the liver. Inflammation may be a very important cause of hepatic steatosis. Elevated pro-inflammatory cytokines can cause M1 polarization of Kupffer cells, which leads to further release of TNFα, IL-1β, and IL-6 [94]. These pro-inflammatory cytokines contribute to hepatic steatosis by activating SOCS, which not only interfere with hepatic insulin signaling as described above, but also inhibit STAT signaling, enhancing expression of the sterol regulatory element binding protein 1 (SREBP-1c) [95]. Despite the state of insulin resistance, hyperinsulinemia further increases transcription of *Srebp1c* and activates SREBP-1c [96]. Activation of SREBP-1c, a transcription factor that enhances de novo lipogenesis, causes further lipid accumulation [97]. Unexpectedly, liver-specific JNK1-null mice on HFD were not protected from glucose intolerance and hepatic insulin resistance and inflammation compared to WT mice on HFD [98], suggesting that JNK1 activation in hepatocytes might not play a role in obesity-driven hepatic insulin resistance. Interestingly, liver-specific JNK2-null mice on HFD were less obese and showed slightly improved glucose tolerance and hepatic insulin resistance compared to the liver-specific JNK1-null mice and WT mice on HFD, suggesting that JNK2 is the predominant isoform that mediates insulin resistance in the liver [99]. Another study also found that antisense oligonucleotide (ASO)-induced JNK2 knockdown in mice on HFD profoundly reduced blood glucose and significantly increased insulin sensitivity measured by HOMA-IR compared to ASO-mediated JNK1 knockdown and wildtype mice on HFD [100]. However, the double KO of JNK1 and JNK2 is even more protective against insulin resistance than the single JNK2 KO [99]. Part of this protection may be mediated by increased FGF21 secretion, as double JNK1 and JNK2 KO upregulated the mRNA expression of FGF21. FGF21 can enhance whole body insulin sensitivity by increasing adiponectin and energy expenditure [101,102]. With regard to the role of JNK in hepatic steatosis, hepatocyte-specific JNK1-null mice developed hepatic steatosis and had significantly higher *Srebp-1* expression compared to wild-type mice [98]. However, these mice were not subjected to HFD, which would closely mimic the development of hepatic steatosis in humans. In contrast, Singh et al. administered an antisense oligonucleotide to knock down JNK1 in a model of established hepatic steatosis and found that this was able to significantly reduce hepatic triglyceride content, whereas JNK2 knock down was not effective [100]. Other studies have suggested a causal role of JNK in HFD-induced steatosis [103,104,105].

### 4.5. Brain

Insulin and leptin are the two main hormones that are necessary in maintaining normal body weight and glucose homeostasis, as they bind to receptors in the hypothalamus [106]. The accumulation of fat, especially long chain saturated FFA, within the hypothalamus has been shown to impede leptin and insulin signaling. This shifts the balance between energy intake and expenditure, towards increased food intake and energy storage [107]. High fat feeding increased JNK activation in both hypothalamic and pituitary neurons. The ablation of JNK1 in the central nervous system (CNS) reduces body weight, increases insulin sensitivity in the CNS, and neuron-specific JNK1-null mice are protected from HFD-induced glucose intolerance and insulin resistance. Interestingly, these mice had increased IL-6 expression in liver, reducing gluconeogenesis and increasing insulin sensitivity [106], most likely due to enhanced insulin sensitivity within AgRP neurons, which causes increased IL-6 in the liver, as described previously. Phenotypically, neuron-specific JNK1-null mice have a reduction in food intake, increased locomotive activity and energy expenditure. This increase in energy expenditure due to JNK1 ablation in neurons is partially explained by activation of the hypothalamic-pituitary-thyroid axis: these mice have increased expression of thyroid hormone-induced genes, as well as elevated levels of T3 and T4 in blood [108]. In a follow-up study to investigate the effects of HFD-induced JNK activation on the hypothalamic-pituitary-thyroid axis, the authors generated mice that have JNK1 and JNK2 ablation specific to the anterior pituitary gland, the source of TSH. It was observed that pituitary-specific JNK1 and JNK2 ablation is required to protect mice from HFD-induced obesity, and glucose and insulin intolerance. In addition, pituitary-specific JNK1 and JNK2 ablation is required to increase TSH and thyroid hormone release, and thyroid hormone-induced gene expression in liver and adipose tissue [109]. 

## 5. Role of JNK in Insulin Secretion

Excess FFA and inflammatory cytokines not only have effects at the level of insulin resistance, but can also contribute to β-cell failure [110]. One aspect of T2D is insulin resistance, but the other lies in defective insulin secretion. In the β-cell, the physiological roles of JNK include mediating cell proliferation and differentiation and mounting the appropriate cellular response to stress. However, the sustained activation of JNK in β-cells, especially during obesity where FFA and pro-inflammatory cytokines are elevated in plasma, can cause β-cell dysfunction and death (Figure 2). The sustained activation of JNK can reduce insulin signaling and secretion within the β–cell [111]. Prolonged activation interferes with the insulin signaling cascade, which involves the activation of IRS1/2, PI3K/AKT pathway, and downstream effector proteins. Decreased tyrosine phosphorylation and increased serine phosphorylation of IRS1/2 will inhibit its activation, and thereby hinder downstream activation of PI3K/AKT pathway [112]. Reduced AKT activation will decrease the degradation of transcription factor FOXO1, allowing for nuclear translocation of FOXO1. Nuclear localization of FOXO1 will prevent PDX1, the transcription factor required for insulin gene transcription [113], from entering the nucleus. In addition, JNK1 can increase nuclear translocation of FOXO1 by direct FOXO1 phosphorylation. Additionally, JNK1/2 can phosphorylate its downstream target, transcription factor c-Jun, to inhibit insulin gene transcription, by inhibiting the enhancer of the insulin gene [114]. In addition, reduced PI3K/AKT activation can inhibit the mTOR pathway, which is required for proinsulin translation [115]. Thus, the activation of JNK1/2 within β-cells reduces the amount of insulin produced. In addition, JNK plays a role in mediating β-cell death: some of the downstream targets of activated JNK are pro-apoptotic factors that can cause the release of cytochrome C from the mitochondria into the cell to induce apoptosis. Furthermore, JNK1/2 plays a role in the production of pro-inflammatory cytokines such as IL-1β, which also mediates cytokine-induced ß-cell death [111].

β-cell specific constitutively active JNK mice display glucose intolerance, decreased insulin secretion and perturbed insulin signaling within β-cells [111]. Although PDX1 is important for insulin gene transcription, PDX1 also upregulates other genes, such as *Glut2* and *glucokinase* [116]. Decreasing the expression of these genes would impact the ability for β-cells to uptake glucose and accordingly respond to plasma glucose levels, possibly explaining the effects observed in mice with β–cell specific constitutively active JNK [111]. IL-1β and TNFα that are locally produced in states of excess FFA and glucose can bind and activate their receptors on β-cells, eventually activating JNK [117]. Increased circulating cytokines can theoretically do the same, although this has not been demonstrated yet. Islets isolated from patients with T2D have elevated pro-inflammatory cytokine expression including IL-1β and TNFα, and chemokine expression such as CCL2 and CCL13 [118,119]. Studies in the INS-1β -cell line with shRNA expression for each JNK isoform revealed that JNK1 is the predominant isoform that plays a role in cytokine-induced β-cell dysfunction, and the knockdown of JNK1 attenuated IL-1β-induced apoptosis [120]. Additionally, the activation of JNK1/2 can inhibit anti-apoptotic factors in the β-cell such as Bcl-2 and Bcl-xL, and also increase pro-apoptotic factors to induce cytochrome C release, ultimately causing β-cell apoptosis [121]. In contrast to the deleterious effects of JNK1/2, JNK3 has been shown to protect β-cells from cytokine-induced apoptosis by increasing IRS2 and AKT2 transcription and expression [122]. Both IRS2 and AKT2 are important for β-cell growth and survival [123]. 

There are major similarities between inflammation in pancreatic islets and in the adipose tissue, especially in terms of the role that macrophages play in mediating the inflammatory response during obesity. There is macrophage accumulation in islets of people with T2D [124,125] and in diet-induced obesity in animals [124,126]. Eguchi et al. showed that M1 macrophages increased in islets of palmitate-infused mice and expression of *Il1b* and *Tnf* was significantly higher in these islets compared to controls, strongly suggesting that macrophages play a role in lipid-induced ß-cell failure. This was confirmed by studies with clodronate, which demonstrated that macrophage depletion ameliorated β-cell dysfunction in palmitate-infused mice and in mouse models of T2D [127]. In addition to macrophage effects on ß-cell, it cannot be excluded that insulin action in the brain regulates β-cell function [128,129]. Hypothalamic inflammation mediated by JNK activation might contribute to the dysregulation of insulin secretion [130,131]. It is the progression into β-cell failure that causes the transition into T2D, as the β-cells are no longer able to compensate for insulin resistance by increasing insulin secretion, and β-cell JNK1/2 are one of the key players in causing such a defect in compensation.

## 6. Role of JNK in Lipotoxicity

**Figure 3:**
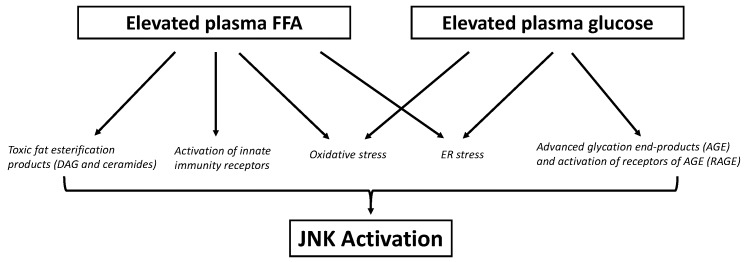
Various mechanisms have been proposed to cause JNK activation during obesity and T2D. Both elevated plasma free fatty acids (FFA) and glucose can accumulate and negatively impact tissues (effects known as lipotoxicity and glucotoxicity, respectively), via JNK activation. Excess FFA can overload the oxidative capacity of cells, leading to incomplete oxidation of FFA, which not only generates reactive oxygen species (ROS), but also causes accumulation of lipotoxic esterification products such as diacylglycerol (DAG) and ceramides. DAG can activate JNK via PKC and ROS, while ceramides can activate JNK via ASK1 or via inhibiting sarco(endo)plasmic reticulum Ca^2+^-ATPase pump (SERCA) to induce ER stress. Saturated FFA can reduce membrane fluidity, which inhibits SERCA to cause ER stress, which in turn activates JNK via IRE1α. Saturated FFA can also activate JNK through receptors of the innate immune system, such as TLRs and NOD receptors. Excess glucose can similarly overload mitochondrial oxidative capacity, which elevates ROS production. Glucose can also undergo non-enzymatic glycosylation reactions to form advanced glycation end products (AGE), and this process also produces ROS. AGE can bind to receptors of AGE (RAGE) to initiate inflammatory processes. Both oxidative stress and AGE can induce ER stress, and ultimately activate JNK.

Lipotoxicity is defined as the negative impact that excess FFA or lipid metabolites exert on tissues such as liver, muscle, ß-cell [132], and hypothalamus [133]. As explained above, there is excess plasma FFA during obesity. The two most commonly elevated FFA are oleate and palmitate, which are unsaturated and saturated FFA, respectively [134]. The exact mechanisms behind FFA-induced impairment in each tissue are still being elucidated, but several candidates have been proposed, including mitochondrial dysfunction and oxidative stress, ceramide synthesis, ER stress, and innate immunity. All these processes can cause the activation of inflammatory kinases, including JNK (Figure 3).

Elevated plasma lipids during obesity, as discussed above, can cause the buildup of fat in organs other than adipose tissue. When mitochondria are no longer able to handle the excess load of FFA, incomplete oxidation of FFA occurs and generates oxidative stress [135]. Fat metabolites within the cells, such as diacylglycerol (DAG), can activate protein kinase C (PKC), which can then activate NADPH oxidase. This enzyme produces superoxide molecules, or ROS, in the cytosol. The generation of ROS can activate JNK in a redox sensitive manner [136]. One of the best characterized MAP3K that responds to ROS is ASK1. This kinase is inhibited by a protein called thioredoxin (Trx) that contains a redox site. ROS oxidizes this site and cause the dissociation of Trx from ASK1. Once dissociated and active, ASK1 will activate JNK [137]. In addition, when JNK is inactive, glutathione S-transferase pi (GSTpi) is bound to JNK to prevent activation. However, the presence of ROS causes the oligomerization of GSTpi and dissociation from JNK, allowing for JNK to be activated. This mechanisms seems to be independent of the classical ASK1/MKK7 pathway for activating JNK [138]. 

Other lipotoxic metabolites are ceramides. The main mechanism of ceramide-induced JNK activation is probably unknown, however there are a few theories. Firstly, ceramides can induce the expression of thioredoxin-interacting protein (TXNIP) in β-cells through an NFκB-dependent mechanism, and TXNIP translocates into the mitochondria to inhibit Trx [139]. By inhibiting Trx, TXNIP will dissociate it from ASK1 and cause JNK activation, as described previously [137,140]. At the same time, TXNIP inhibition of Trx, which is an antioxidant enzyme, will promote the ROS-mediated activation of the intrinsic mitochondrial apoptotic pathway to cause β-cell death [141]. Secondly, ceramides might be able to block sarco(endo)plasmic reticulum Ca^2+^-ATPase pump (SERCA), causing a reduction in ER Ca^2+^ levels, thus inducing ER stress [142]. Lastly, ceramides can activate Rac-1, PKCζ, and TAK-1 to ultimately activate JNK [143].

Another mechanism involved in lipotoxicity is endoplasmic reticulum (ER) stress. The ER is responsible for ensuring proper protein maturation and folding, and anything that can disrupt the homeostasis within the ER can cause ER stress. Prolonged exposure to saturated FFA such as palmitate can disrupt intracellular Ca^2+^ regulation [144]. Saturated FFA incorporation into membranes reduces fluidity, which can inhibit transmembrane proteins such as SERCA [145], impacting Ca^2+^ shuttling back into the ER [135,146]. The reduction in fluidity can also increase ER stress markers independent of SERCA [147,148]. ER stress activates the unfolded protein response (UPR), whose end result is either the recruitment of additional chaperone proteins to mitigate the stress or the induction of apoptosis. The markers of UPR include ER proteins PERK, IRE1α and ARF6, all of which are elevated in T2D. The activation of JNK is downstream of IRE1α to promote apoptosis [149]. IRE1α, when active, associates with TNFR-associated factor 2 (TRAF2), an adaptor protein, to activate the MAP3K ASK1, and ultimately JNK [144,150,151,152,153].

Recent studies have established the role of the innate immune system in mediating lipotoxicity induced by saturated fat. Saturated FFAs can activate TLR2 and TR4, most likely indirectly via associating with proteins such as fetuin-A [154] or high-mobility group box 1 (HMGB1) [155]. TLR2 [156] and 4 [127,157] have been implicated in fat-induced hepatic and peripheral insulin resistance and β-cell dysfunction. JNK and IKKβ activation is downstream of TLR activation, via TAK1 [158]. Another class of receptor of the innate immune system is the nucleotide-binding oligomerization domain (NOD) receptors, which unlike the TLRs, are situated within the cytoplasm. This allows for sensing of intracellular abnormalities, in contrast to TLRs sensing extracellular abnormalities [159]. It seems that similar to the TLRs, saturated FFA but not unsaturated FFA are able to activate NOD receptors, which signal via RIPK2, to also activate JNK and IKKß [160]. These receptors are capable of mediating HFD-induced insulin resistance [161] and our group is currently implicating them in ß-cell dysfunction. Other innate immune receptors that are activated by FFA, likely via ROS, are the nucleotide-binding domain leucine rich repeat (NLR)-forming inflammasomes, including NLR and pyrin domain containing receptor 3 (NLRP3). These have been implicated in both fat-induced insulin resistance and ß-cell dysfunction [162,163,164]. They act as a platform for cytokine maturation, which ultimately involves the activation of JNK by their receptors.

### 6.1. Skeletal Muscle Lipotoxicity

The abnormal buildup of fat in skeletal muscle is associated with insulin resistance. As indicated above, mitochondrial dysfunction is a key contributor of fat buildup in skeletal muscle and consequent lipotoxicity. Mitochondrial dysfunction, as well as the activation of NADPH oxidase by PKC, increases oxidative stress within myocytes [165], which can activate JNK. PKCs can cause insulin resistance directly by phosphorylating Ser/Thr residues on IRS proteins [166]. In addition, PKC can phosphorylate JNK via MKK4/7 [17]. Traditionally, muscle seems to be relatively spared from ER stress compared to liver and pancreatic islets [167], however palmitate treatment of C2C12 muscle cells increased UPR markers [168]. Ceramides also impact insulin signaling via activating JNK and independent of JNK via activating a phosphatase, as seen when cultured myotubes exposed to palmitate showed a reduction in AKT activation, due to dephosphorylation at Thr308 [169,170,171]. In addition, mice on a high-fat diet for six weeks showed upregulation of ER stress and UPR markers in the soleus and tibialis anterior [168], suggesting that ER stress is involved in the mechanism by which excess FFA causes skeletal muscle lipotoxicity [168], potentially via JNK. With respect to inflammation, TLR4 and TLR2 activation by saturated FFA also causes insulin resistance, potentially via JNK in skeletal muscle [157]. Lipotoxicity not only affects skeletal muscle but also cardiac muscle, as described under Section 8.

### 6.2. Hepatic Lipotoxicity

Similar mechanisms are implicated in fat induced hepatic insulin resistance as those described above for muscle insulin resistance, i.e., PKC activation, oxidative stress and activation of inflammatory kinases [172,173,174,175]. Ceramides have been implicated in some studies [176,177]. ER stress activation of JNK is an important mechanism of insulin resistance induced by fat [167,178]. p38 is activated as well, via TRAF [103] or PKC [179], and together with JNK and PKC can contribute to both insulin resistance and hepatic steatosis [103,173,180,181], although there is some controversy about the p38 effect on hepatic lipid metabolism [182]. Lipid accumulation occurs despite insulin resistance because of lipid overload and inflammation. Cytokines induce hepatic and peripheral insulin resistance in part via SOCS, which antagonize the insulin receptor mediated phosphorylation of IRS1/2 [80] and can increase hepatic fat accumulation via antagonizing STAT3-mediated inhibition of the lipogenic transcription factor SREBP-1c [95]. Innate immunity receptors potentially activating JNK participate in hepatic lipotoxicity, as shown by protection from hepatic insulin resistance by TLR2 [183] or 4 KO [184]. JNK activation by excess FFA not only causes insulin resistance in the liver, but also liver injury [88]. In addition to oxidative stress and ER stress, several oxidative stress/ER stress-independent mechanisms of JNK activation have been described in the context of saturated FFA-induced hepatocyte death in in vitro studies. Saturated FFA can cause Cdc42/Rac1, a small membrane-bound GTPase, to interact with a MAP3K called MLK3 [88]. This will eventually lead to JNK activation and cause pro-apoptotic factors such as Bax to be activated. Next, glycogen synthase kinase (GSK3α/ß) also plays a role in this pathway, presumably downstream of MLK3. It has been suggested that GSK3ß interacts with certain MAP3Ks, such as MLK3 or MEKK1, to enhance JNK activation [185]. Interestingly, FFA-induced cell death was only reduced in primary hepatocytes isolated from JNK2-null mice and not JNK1-null mice, suggesting that JNK2 plays a larger role in the liver in mediating FFA-induced hepatocyte death in vitro [186].

### 6.3. β-Cell Lipotoxicity

Intra-islet fat accumulation can severely impact β-cell function and mass, which negatively impacts the ability of β-cells to compensate for insulin resistance [7]. PKC and ROS have been implicated in both β-cell dysfunction and death [187,188,189]. The ROS generated upon FFA exposure are lipid peroxides and extramitochondrial superoxide produced by PKC-stimulated NADPH oxidase [190,191]. The β-cell is very sensitive to oxidative stress because of its low content of antioxidant enzymes [192]. ROS generation is induced with both saturated and monounsaturated fat. Although oxidative stress can activate JNK, there seems to be differential activation of JNK in β-cells between monounsaturated and saturated fat. Islets treated with a JNK1 peptide inhibitor (D-JNKi) and islets isolated from JNK1-null mice were protected from palmitate-induced β-cell dysfunction [193], but JNK1 inhibitors did not protect islets from oleate-induced β-cell dysfunction [144]. This is likely because the main JNK activators during saturated FFA exposure are ER stress, ceramides and innate immunity receptors, which are induced/stimulated mainly or exclusively by saturated fat. In β-cells, the ER is important in ensuring that insulin folds properly [149]. Thus, β-cells are especially sensitive to ER stress, and JNK stimulation by ER stress accounts for palmitate–induced IRS-1 serine phosphorylation [167], reduced insulin gene transcription [167], and apoptosis [144]. Ceramides are also involved in reducing insulin gene transcription independent of JNK [194,195,196,197]. As described above for insulin resistance, β-cell dysfunction by saturated fat was prevented in TLR2- [156] and TLR4-null mice [127,198], and in NLRP3-null mice [162,163,164].

### 6.4. Hypothalamic Lipotoxicity

As mentioned previously, high-fat feeding has been found to cause hypothalamic inflammation resulting in weight gain and insulin resistance. Interestingly, inflammation within the hypothalamus from HFD is an acute effect; it is seen after just several days of HFD. This is in contrast to peripheral inflammation caused by HFD that is observed in a more chronic setting [199]. Intracerebroventricular (ICV) administration of the JNK inhibitor SP600125 in mice on HFD reversed HFD-induced insulin resistance in the hypothalamus. There was an increase in insulin-induced activation of IRS and AKT [200]. The genetic activation of JNK in AgRP neurons caused weight gain and adiposity, also suggesting a causal role of JNK in hypothalamic inflammation [201]. In addition, direct ICV administration of palmitate significantly elevated pro-inflammatory cytokines TNFα and IL-1β, phosphorylation of IKKβ, and blunted insulin-induced activation of AKT in the hypothalamus, suggesting that elevation of saturated FFA within the brain can induce hypothalamic inflammation and insulin resistance [202,203]. Similar mechanisms have been implicated in hypothalamic JNK activation by FFA as those described in other tissues: long-chain saturated FFAs can activate TLR4 within the hypothalamus and cause JNK activation [204]. One in vitro study also found that when hypothalamic cells were exposed to palmitate, JNK activation was induced by the inhibition of AMPK, and JNK in turn induced ER stress. The latter mechanism was not explored but might be related to the JNK activation of inflammatory cytokines and oxidative stress [205]. The induction of ER stress with thapsigargin administered ICV induced both leptin and insulin resistance in the hypothalamus, which caused increased weight gain and food intake. In addition, hypothalami of mice on HFD had significantly elevated markers of ER stress and JNK activation compared to mice on a low-fat diet (LFD). Introduction of PBA treatment (chemical chaperone) into mice on HFD significantly reduced food intake, demonstrating that alleviating ER stress within the hypothalamus can reduce leptin resistance in the context of HFD-induced hypothalamic inflammation [206]. Ceramides administered centrally also increase ER stress and induce weight gain in mice. In this model, centrally administered ceramide also induced expression of pro-inflammatory markers such as IL-6, IKKβ and TNFα [207]. It must be mentioned that unsaturated FFA, such as linolenic and oleic acid, reduce JNK activation in the hypothalamus in the context of saturated FFA-induced hypothalamic dysfunction [208].

## 7. Role of JNK in Glucotoxicity

In T2D, especially during chronic and uncontrolled hyperglycemia, elevated glucose can have impairing effects on insulin-sensitive and insulin secreting tissues, termed glucotoxicity [209]. Excess glucose can overload the electron transport chain responsible for oxidative phosphorylation in mitochondria, elevating mitochondrial superoxide production [210]. Glucose can also activate PKC and NADPH oxidase, elevating non-mitochondrial superoxide production [211,212,213]. In addition, glucose can undergo non-enzymatic glycosylation reactions to form advanced glycation end products (AGE), in which ROS are produced [214]. AGE can interact with receptors of AGE (RAGE), initiating inflammation. Elevation of ROS and non-enzymatic glycosylation of proteins by elevated glucose can also induce ER stress [215,216,217,218]. Downstream of all these processes is the activation of inflammatory kinases, including JNK (Figure 3). 

### 7.1. Adipose Tissue Glucotoxicity

Chronically elevated glucose can increase oxidative stress [219,220,221] and ER stress within adipocytes [222,223], both of which can induce JNK activation [167] and ultimately result in adipocyte insulin resistance [224,225]. The production of pro-inflammatory cytokines contributes to a feed-forward cycle, increasing JNK activation and insulin resistance, since cytokines can recruit immune cells such as macrophages, and would further propagate inflammatory effects on adipose tissue [222]. However, hyperglycemia-induced insulin resistance in adipocytes might be mediated primarily by PKCζ, as there was no difference in IRS1 Ser307 phosphorylation status between primary adipocytes isolated from control and glucose-infused rats [226]. 

### 7.2. Skeletal Muscle Glucotoxicity

Hyperglycemia-induced insulin resistance in skeletal muscle was found to be mediated by oxidative stress. Specifically, high glucose caused the elevation of protein carbonyl, a marker of skeletal muscle oxidative stress, in skeletal muscle in rats infused with glucose for 6 h and antioxidant treatment reversed muscle insulin resistance [227]. Although in vitro studies in L6 muscle cells found that hydrogen peroxide caused JNK activation [228], JNK1-null mice were not protected from peripheral insulin resistance induced by glucose infusion, suggesting that other mechanisms are involved [229].

### 7.3. Liver Glucotoxicity

Chronic hyperglycemia can cause insulin resistance in hepatocytes [230]. The contribution of JNK in mediating the toxic effects of elevated glucose is similar to that of hepatic lipotoxicity: elevated oxidative stress [231] can activate JNK to cause insulin resistance and hepatocyte death [232]. In addition, the effect of hyperglycemia on the liver is intimately related to lipotoxicity, as chronic sugar exposure, whether it be glucose or fructose, can increase fatty acid accumulation in the liver. Fructose is especially lipogenic as it enters glycolysis beyond the gateway keeper glucokinase enzyme [232].

### 7.4. β-Cell Glucotoxicity

Exposure to elevated glucose can lead to the deterioration of β-cell function and β-cell death. Similar to how elevated palmitate causes the inhibition of SERCA, chronically elevated glucose also reduces the expression of SERCA in β- cells, thus playing a role in inducing ER stress [146]. In addition, glucose can induce IL-1β expression and secretion in islets, which will propagate the feed-forward cycle of pro-inflammatory cytokines and its effects on macrophages and β-cells [119]. Furthermore, mitochondrially produced ROS are particularly effective in inducing ER stress [233,234], as they communicate the redox changes to the ER [234]. Oxidative stress has been implicated in glucose-induced β–cell dysfunction in in vivo models by us and several other groups [233,235,236], and our group found that JNK1 plays a causal role in β-cell dysfunction specifically induced by glucose [229]. Our group has shown that glucose-mediated β-cell dysfunction can also involve ER stress. In vivo infusion of glucose for 48 h in rats elevated mitochondrial superoxide generation in addition to ER stress markers PERK, IRE1α and ATF6. Co-infusion of glucose with PBA, a chemical chaperone that assists in protein folding and thus reduces ER stress, reduced IRE1α activation and oxidative stress in vivo, and prevented glucose-induced ß-cell dysfunction similar to superoxide dismutase mimetics and JNK inhibitors [234]. 

### 7.5. Hypothalamic Glucotoxicity

Although the role of dietary sugars on hypothalamic inflammation has not been as extensively studied as the role of lipids, Gao et al. recently demonstrated that dietary sugars can induce hypothalamic inflammation. This group found that there was significantly increased AGE within the arcuate nucleus of the hypothalamus in mice on a high-carbohydrate high-fat (HCHF) diet compared to mice given a low-carbohydrate high-fat (LCHF) diet [237]. Although neuron specific JNK-1 null mice have reduced weight gain and insulin resistance, and glucotoxicity is a potent activator of JNK, the specific role of JNK1 in glucotoxicity at the level of hypothalamus, to our knowledge, has not been addressed. 

## 8. Role of JNK in Diabetic Complications

Chronic hyperglycemia can cause various complications such as retinopathy, neuropathy and nephropathy via the glucotoxicity mechanisms discussed above, including oxidative stress. Increased plasma glucose can lead to the formation of AGEs, and these products can bind to RAGE to increase oxidative stress. Retinal endothelial cells exposed to high glucose concentrations had elevated oxidative stress and activation of JNK [238,239], however these studies did not evaluate whether JNK caused cell death. In diabetic neuropathy, high glucose and pro-inflammatory cytokines can cause excessive oxidative stress in neurons leading to neuronal death. The effects of ERK and p38 kinases in the development and maintenance of pain are well studied, however not much is known about how and whether JNK does the same. JNK activation in neurons leads to cell death, and JNK inhibition by SP600125 demonstrated benefits in pain response in rodents [240]. Similarly, oxidative stress due to chronic hyperglycemia can cause mitochondrial dysfunction in glomerular cells of the kidney, leading to the development of diabetic nephropathy [241,242]. Patients with this diabetic complication usually had elevated renal JNK activation when kidney samples were biopsied [243,244]. The activation of JNK within the kidney can cause local production of pro-inflammatory cytokines that would further propagate kidney inflammation [245]. In addition, macrovascular complications such as atherosclerosis are commonly observed in patients who are obese or have T2D [246]. The activation of JNK in vascular endothelial [247,248] and smooth muscle cells [249] can trigger the release of pro-inflammatory cytokines, as well as promote plaque formation within blood vessels [246]. Similar to skeletal muscle lipotoxicity, there is also lipotoxicity of cardiomyocytes which is found in diabetic cardiomyopathy. Palmitate-induced apoptosis in cardiac myocytes was associated with elevated oxidative stress and the activation of JNK, along with ERK1/2 and p38 [250], and the knockdown of JNK1 with an ASO partially prevented ceramide-induced cardiomyocyte apoptosis [251]. This suggests that JNK1 may be important for saturated FFA-induced cardiac dysfunction.

## 9. JNK as a Therapeutic Target

Despite the evidence that inflammation plays an important role in metabolic disease, current anti-inflammatory therapy has failed to produce substantial results [252,253,254,255], which is why research into new inflammatory targets is warranted. Although it is unlikely that targeting JNK alone would prove to be an effective treatment of human T2D, which is a more complex disease than T2D in animals, there is substantial preclinical evidence that suggests JNK is a promising therapeutic target in the context of obesity and T2D (perhaps for combination treatment against multiple inflammatory targets, and perhaps for the prevention of T2D in insulin resistant states). Genetic knockouts of JNK1 in metabolic studies have shown benefits, including protection against obesity-induced insulin resistance. These studies have also provided a valuable insight into the role of JNKs in various metabolically active tissues. There are several JNK inhibitors that have been studied up to now: ATP-competitive inhibitors and small peptide inhibitors (Table 1).

The ATP-competitive inhibitors that have been used in JNK-related studies are mainly SP600125 and CEP-1347. Both of these molecules occupy an ATP-binding site: SP600125 directly inhibits JNK [256], while CEP-1347 inhibits MLK [257], the MAP3K upstream of JNK. Binding of the ATP-binding site prevents phosphorylation and activation of JNK and MLK, respectively. Although drug screening has identified several more molecules that have higher JNK isoform or pathway specificity, efficacy of this class of inhibitor can be reduced by high levels of ATP. The use of SP600125 has been limited to pre-clinical studies due to the disadvantages that come with its usage. Firstly, it inhibits all isoforms of JNK, which is not desired as different diseases implicate different isoforms of JNK [256]. As mentioned earlier, not all isoforms of JNK play a detrimental role. JNK3 seems to play a protective role in ß-cell function, especially against cytokine-induced cell death [122]. Additionally, at high doses of this compound, non-specific effects arise, such as inhibition of other kinases (i.e. the MAP2Ks MKK3/4/6/7). Although these off-target effects might contribute to the benefits observed from using this compound, their presence also demonstrates the lack of specificity of this molecule [256]. CEP-1347 had promise in pre-clinical studies in protecting against Parkinson’s disease; however, it was stopped in Phase II/III clinical trials due to lack of effect in delaying the onset of Parkinson’s-related disabilities [258].

Most recently, a phase II clinical trial was completed with another ATP-competitive JNK inhibitor called AS601245, or Bentamapimod [259]. This compound has an IC50 of 80nM for JNK1, 90nM for JNK2, and 230nM for JNK3 [260], suggesting some specificity for JNK1/2 over JNK3. Bentamapimod showed promise in preclinical studies in models of neuronal cell death and cancer. In most tumor cell lines, there is overactivation of JNK that could contribute to proliferation, therefore the development of a JNK inhibitor could benefit specific cancers [261]. Although the above-mentioned clinical trial studied the effects of Bentamapimod on endometriosis, which is a disease that involves cell proliferation (results were not published), it would be interesting to know whether the treated subjects displayed improved metabolic function. 

The peptide inhibitors of JNK are more specific to JNK, as they specifically target the JNK binding domain (JBD) that will inhibit JNK activation. One example of this class of inhibitor is the D-JNKI-1 peptide, which can penetrate the cell. This peptide inhibitor has shown protective effects in neurons by preventing c-Jun activation [256]. It was demonstrated that this peptide is safe for administration, and well tolerated, in humans [262]. In fact, this peptide, also called XG-102 or AM-111, has been evaluated in phase III clinical studies for inflammation and pain post-surgery, and hearing loss, however results have not been published yet. Similar to Bentamapimod, it would be interesting to know whether the use of this peptide improves metabolic function in the treated subjects.

Other peptide inhibitors of JNK closely resemble the JNK interacting protein (JIP), a scaffolding protein, providing a promising method for more specific JNK inhibition. These inhibitors are able to exert neuroprotective effects by preventing neuronal apoptosis [263]. Another peptide inhibitor is the TAT-JIP protein, which was shown to also specifically inhibit JNK activity, and was able to reduce cytokine production in human T lymphocytes [264]. This peptide demonstrated beneficial effects on glucose tolerance and insulin sensitivity in mice, and also upregulated insulin signaling in liver, adipose tissue and muscle [265]. However, to our knowledge, the TAT-JIP protein has not been used in clinical trials.

## 10. Conclusions

There is no question that obesity poses a major risk factor for the development of T2D, but what is still uncertain are the processes in which these disorders are linked. The inflammatory kinase JNK is proposed to be a key player, as whole-body JNK1-null mice protect against obesity-induced insulin resistance. Tissue-specific ablation of JNK1 can also play a protective role, to a varying extent. Regardless of the mechanism behind how cytokines, elevated FFA and hyperglycemia activate JNK, this presents a potential therapeutic target to restore insulin sensitivity and preserve β-cell function.

## Figures and Tables

**Table 1:** Summary table of JNK inhibitors that have been studied. There are two main classes of inhibitors: the ATP-competitive inhibitors, and the peptide inhibitors of JNK.

Class	Drug	Mechanism of Action	Summary
ATP-competitive inhibitors	SP600125	Directly inhibits all JNK isoforms at the ATP-binding site	Investigation of this compound is limited to pre-clinical studies
	CEP-1347	Inhibits MLK at the ATP-binding site	Stopped in Phase II/III clinical trials; was developed for Parkinson’s disease
	AS601245 (Bentamapimod)	Directly inhibits all JNK isoforms at the ATP-binding site, but with higher selectivity for JNK1 and JNK2	Phase II clinical trial recently completed about the effects on endometriosis
Peptide inhibitors of JNK	D-JNKI-1 peptide (XG-102/AM-111)	Specifically targets the JNK binding domain (JBD) to inhibit JNK activation	Phase III clinical trial recently completed about the effects on inflammation, post-operative pain, and hearing loss
	TAT-JIP protein	Resembles JNK interacting protein (JIP) to inhibit JNK	Improves glucose tolerance and insulin sensitivity in mice and upregulates insulin signaling in insulin-sensitive tissues. No available data in humans
